# Interspecific interactions explain variation in the duration of paternal care in the burying beetle

**DOI:** 10.1016/j.anbehav.2015.08.014

**Published:** 2015-11

**Authors:** Ornela De Gasperin, Ana Duarte, Rebecca M. Kilner

**Affiliations:** Department of Zoology, University of Cambridge, Cambridge, U.K.

**Keywords:** interspecific interactions, mites, *Nicrophorus*, paternal care, phoresy

## Abstract

Why is there so much variation within species in the extent to which males contribute to offspring care? Answers to this question commonly focus on intraspecific sources of variation in the relative costs and benefits of supplying paternal investment. With experiments in the laboratory on the burying beetle, *Nicrophorus vespilloides*, and its phoretic mite *Poecilochirus carabi*, we investigated whether interactions with a second species might also account for intraspecific variation in the extent of paternal care, and whether this variation is due to adaptation or constraint. In our first experiment we bred beetles in the presence or absence of phoretic mites, using a breeding box that mimicked natural conditions by allowing parents to leave the breeding attempt at a time of their choosing. We found that males abandoned their brood sooner when breeding alongside mites than when breeding in their absence. Female patterns of care were unchanged by the mites. Nevertheless, in this experiment, no correlates of beetle fitness were affected by the presence of the mites during reproduction (neither paternal life span after reproduction nor brood size or average larval mass). In a second experiment, we again bred beetles with or without mites but this time we prevented parents from abandoning the brood. This time we found that both parents and the brood suffered fitness costs when breeding alongside mites, compared with families breeding in the absence of mites. We conclude that males adaptively reduce their contributions to care when mites are present, so as to defend their offspring's fitness and their own residual fitness. Interspecific interactions thus account for intraspecific variation in the duration of paternal care.

## Highlights

• The extent of paternal care varies greatly within species.
• Some of this variation might be due to interspecific interactions.
• We investigated how mites influence paternal care in the burying beetle.
• We found that males leave their brood earlier when mites are present.
• We show that this is adaptive because it enhances offspring fitness.

The extent to which males care for their young varies considerably within species, commonly to a greater extent than is typically seen among females (e.g. Sheldon, 2002) and for a range of reasons that are not mutually exclusive. For example, some of this variation has been attributed to individual variation in the optimal level of paternal care (e.g. Møller and Birkhead, 1993, Balshine-Earn et al., 1998, Neff, 2003, Manica, 2004, Velando et al., 2006, Ward et al., 2009). In species in which either sex can successfully raise offspring singlehandedly, or in which there is biparental care, variation in paternal care has additionally been explained by interactions with the mother of their offspring. Males may increase their level of care if raising offspring with a female of very high quality (Johnstone and Hinde, 2006, Moreno and Osorno, 2003), because the benefits to be gained are correspondingly greater. Or they may contribute less investment if paired with a high-quality female, because she can more easily bear the greater costs of providing more care (Lessells & McNamara, 2011). Sexual conflict between parents over the division of the costs associated with parental care can further explain patterns of paternal care (Bennett & Owens, 2002). If males win this conflict, they may force the female to bear greater fitness costs as a result of providing care, and so contribute relatively little parental care themselves (Van Dijk, Szentirmai, Komdeur, & Székely, 2007). However, if females gain the upper hand, males may take the greater share of the fitness costs associated with parental care and contribute to a far greater extent than the female (Lessells, 2012).

Here we consider whether interactions with other species might also influence the extent of paternal care. Interspecific interactions can strongly impact the reproductive success of fathers and their young. They can reduce the size of the brood (Clotfelter and Yasukawa, 1999, Hillegass et al., 2010), increase the success of individual offspring by increasing individual size (Fredensborg and Poulin, 2006, Shostak, 2009) and/or reduce (Watson, 2013) or increase the life span of fathers (Hurd, Warr, & Polwart, 2001). Moreover, interspecific interactions are already known to change paternal contributions to offspring care (Smith, 1980, Dewsbury, 1985, Zeh and Smith, 1985, Smith and Wootton, 1995, Tallamy, 2001, Haley and Müller, 2002, Requena et al., 2009, Grayson et al., 2013). For example, heavily parasitized upland bully males, *Gobiomorphus breviceps*, spend more time fanning their eggs than males that are more lightly infected (Hamilton & Poulin, 1995), while male spotless starlings, *Sturnus unicolor*, adjust their paternal effort in response to the extent of egg spotting, a trait induced by an ectoparasite carried by females (Avilés, Pérez-Contreras, Navarro, & Soler, 2009). However, in most instances it is not known how these changes in paternal behaviour influence the fitness of males. Therefore it is not clear whether changes in the male's contributions to care are imposed by constraints (the male and his offspring could have greater fitness, were it not for costs imposed by a second species) or whether they are adaptive (the change in male behaviour induced by a second species serves to maintain or even enhance the male's fitness and the fitness of his young). We addressed this shortcoming by investigating interactions between the burying beetle, *Nicrophorus vespilloides*, and a phoretic mite commonly associated with it (*Poecilochirus carabi*, Mesostigmata, Parasitidae).

We began by setting up *N. vespilloides* pairs to breed in the laboratory, with and without *P. carabi*, and determined the duration of paternal care in each scenario (experiment 1). After finding that mites did indeed affect the duration of paternal care of the burying beetle, we tested whether these changes were due to adaptation or constraint, by forcing males to remain with their brood for longer than they stayed in the first experiment, again staging breeding events with and without mites (experiment 2). We predicted that if the change in male care induced by mites was adaptive then components of fitness should be smaller in males and their offspring when males bred alongside mites and were forced to remain with the brood until the end of the breeding event than when males were allowed to leave. However, if the duration of care induced by mites was the result of a constraint then mites should consistently reduce components of fitness in the burying beetle, irrespective of the extent of paternal care.

## Methods

### Ethical Note

We gently collected larvae in their dispersal stage from breeding boxes and we placed them in eclosion boxes with moist soil. We gently removed adults at eclosion from these boxes and housed them in small transparent plastic containers filled with moist soil. We provided them with adequate food twice a week until they reached sexual maturation. After each experimental breeding event we returned experimental individuals to our standard laboratory housing conditions. During our experiments we handled our beetles with care and they were not harmed at any stage. None of the beetles that we used showed any signs of stress before, after or during the experiments.

### Study Species

Burying beetles (*Nicrophorus* spp.) use the body of a small dead vertebrate as a resource for reproduction. Together, the two parents remove the fur or feathers, roll the flesh into a ball, smear it with antimicrobials and inter it in a shallow grave (Scott, 1998). During this process of carcass preparation the female lays her eggs in the soil nearby. After hatching, the larvae crawl to the carcass where they take up residence in a specially prepared crater on the ball of flesh. There, they solicit food from their attendant parents, which also defend the offspring and carcass from potential rivals (Scott, 1998). *Nicrophorus vespilloides* males typically stay with the brood for a few days after hatching (Pukowski, 1933), before flying off in search of new mating opportunities, although there can be considerable variation in the timing of the male's departure (Scott, 1998). After the male leaves, the female remains with the brood until approximately 1 week after pairing, at which point the larvae disperse from the scant remains of the carcass to pupate in the soil. Females then depart to find new carrion for reproduction (Pukowski, 1933, Scott, 1998).

Natural populations of burying beetles interact with several species of phoretic mites (Wilson & Knollenberg, 1987). The *P. carabi* species complex comprises several species that are morphologically similar (Müller and Schwarz, 1990, Brown and Wilson, 1992). We focused on the *P. carabi* complex because they are the most common mites we find on naturally caught burying beetles at our field sites (most of the mites found on *N. vespilloides* beetles in nature are *P. carabi* sensu stricto; Schwarz, Starrach, & Koulianos, 1998). These mites are readily apparent as the deutonymphs (the phoretic stage) are large, very mobile and aggregate on the beetle's head and thorax. They use the burying beetle as a means of transport between opportunities for reproduction on carrion. Once a beetle has located a carcass, the mites alight, moult into sexually mature adults and breed. Their life cycle closely matches the duration of parental care and the majority of the next generation of deutonymphs leaves the carcass on the departing parents (Schwarz & Müller, 1992). Experimental studies have revealed a somewhat complex relationship between *P. carabi* and the burying beetle. Some studies suggest that *P. carabi* is beneficial to the burying beetle because the mites help defend the carcass breeding resource from rival species by piercing blowfly eggs, particularly when the carcass is buried shallowly (Springett, 1968, Wilson, 1983, Wilson and Knollenberg, 1987). Furthermore, experiments examining the relationship between the congeneric burying beetle *Nicrophorus orbicollis* and *P. carabi* suggest that mites can provide long-term fitness benefits for burying beetles, although not if the mites are at very high densities (Wilson & Knollenberg, 1987). However, phoretic mites can reduce burying beetle reproductive success, by eating their eggs (Beninger, 1993, Blackman and Evans, 1994). Furthermore, *P. carabi* can reduce components of male and brood fitness, depending on their density on the carcass (De Gasperin and Kilner, 2015, De Gasperin, 2015). Nevertheless, the effect of *P. carabi* on the duration of male care is not yet known.

### General Stock Maintenance

#### The beetle colony

All the beetles used in these experiments came from a stock population initially founded in 2005. Every year new field beetles are brought into the colony between April and September, and bred with our population colony to avoid inbreeding. Before introducing field beetles we removed any mites on them (see below), and thus kept our burying beetle colony separate from our mite colony. All beetles were kept in small plastic containers (12 × 8 cm and 2 cm high) filled with moist soil and fed twice a week with small pieces of minced beef. The colony was maintained in a laboratory at 20 °C and on a 16:8 h light:dark cycle. Adult beetles bred when they were 2–3 weeks old in plastic breeding boxes (17 × 12 cm and 6 cm high) filled two-thirds with moist soil with a mouse carcass. Note that these general methods mean that all the beetles used in the experiments described here developed as larvae in an environment without mites.

#### The mite colony

Deutonymphs of the *P. carabi* species complex were harvested from field-caught *N. vespilloides* by anaesthetizing the burying beetle with CO_2_, and using a brush and tweezers to remove and count the mites. We did not identify individual mites further down to species level, hence we cannot be 100% sure if our mite populations included a mix of *P. carabi* species or a single species. However, *P. carabi* sensu stricto is the most common mite species found in *N. vespilloides* (Schwarz et al., 1998). Since we harvested deutonymphs from field-caught *N. vespilloides*, the mites used in the experiment are representative of the naturally occurring *P. carabi* on our study species.

Once separated from the burying beetle, we kept the mites in plastic containers (17 × 12 cm and 6 cm high) filled with moist soil, and fed them once a week with minced beef. The containers were kept inside cupboards, and one burying beetle lived alongside the mites in each plastic container (this beetle was not used in any experiments). We bred the mite colony once a month. For this, we placed spare pairs of burying beetles from our stock population to breed on the carcass of a mouse and introduced about 15 mites into the breeding box (17 × 12 cm and 6 cm high). At the end of the reproductive event we anaesthetized both beetles and kept the mites that were dispersing on them.

### Measurements of Burying Beetle Fitness

To assess the effect of our treatments on offspring fitness, we counted the larvae produced and we also measured average larval mass at dispersal because this has been shown to be a good predictor of larval performance (Lock, Smiseth, & Moore, 2004). We used adult life span to measure the fitness costs associated with parental care. In nature, *N. vespilloides* is an opportunistic breeder (Schwarz and Müller, 1992, Scott, 1998). Longer life span after reproduction thus enhances lifetime reproductive success in nature because it increases the likelihood of encountering carrion again, which is essential for reproduction, and thus affords more breeding opportunities (Cotter et al., 2010, Cotter et al., 2011, Ward et al., 2009). In addition, males that are unsuccessful at locating carrion may attempt to attract females for mating by secreting pheromones (Eggert and Müller, 1997, Müller et al., 2007, Scott, 1998). Females can store sperm from these matings for use in future reproduction (Müller & Eggert, 1989). In nature, males commonly sire offspring without attending the carcass upon which larvae are raised (Müller et al. 2007). Thus males that live longer can increase their lifetime reproductive success by mating more often and with more females.

### Experiment 1: Do Mites Change Paternal Care Duration?

**Figure 1:**
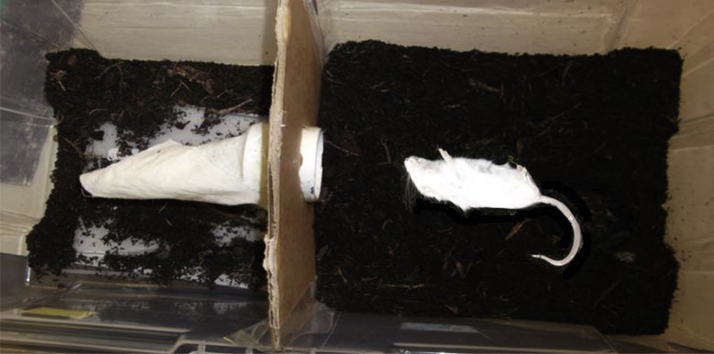
The breeding box used in experiment 1, showing the one-way valve in the cardboard partition through which beetles could leave. Beetles were placed to breed on the right side of the box.

To test whether breeding alongside mites changed the duration of paternal care, we placed pairs of sexually mature, virgin beetles to breed on an 8–15 g dead mouse (mean carcass mass = 11.55 g, SD = 1.80) inside a plastic container (28.5 × 13.5 cm and 12 cm high) with 4 cm of soil. This container was divided in two by a cardboard partition. There was a short tube in the middle of the cardboard which worked as a one-way valve; beetles could go through it to the other side of the breeding box but were unable to return because folds of fabric closed behind them as they left (Fig. 1).

We established two different treatments: beetles breeding with or without phoretic mites. For the ‘with mite’ treatment, we introduced 10 *P. carabi* deutonymphs into the breeding boxes at the same time as we introduced the mouse. Using fine tweezers, we carefully placed the mites on the soil surrounding the mouse. This degree of mite infestation falls within the range brought naturally to the carcass by a pair of burying beetles (8–16 mites per breeding pair, Schwarz & Müller, 1992). Because the carcass is a key resource for both the beetles and mites, we weighed each carcass twice: just prior to giving it to the beetles and after it had been prepared for reproduction but before larval hatching. We weighed carcasses within a 2 h time frame and at a fixed point after the start of the experiment (56 h after pairing). We recorded parental departure time from the carcass by checking each box six times a day (from 0800 to 2000 hours) on each day after pairing the beetles. We collected any beetle that had abandoned the brood: these were individuals that had left the breeding chamber and passed through the one-way valve. We also removed, using tweezers, and counted all the mites dispersing on the beetle. To mimic natural conditions, we did not reintroduce these mites into the breeding box, but instead returned them to the mite stock population. We simulated the removal of mites from beetles dispersing without mites (both from the ‘with mites’ and from the ‘without mites’ treatment) to ensure all beetles were handled to the same extent.

Eight days after the start of the experiment (which is when larvae from *N. vespilloides* usually disperse from the carcass; Scott, 1998) we counted the larvae, weighed the brood, and removed parents that were still on the carcass, taking this date as their departure time from the carcass. By this time, there was only one male that had not abandoned the (bones of the) carcass in all our 91 replicates, and six females that had not left. Furthermore, in all cases there was virtually no meat left on the carcass and the larvae had started to disperse into the soil. We also removed and counted all the mites that each of the parents carried before placing each adult alone in a small plastic box (12 × 8 cm and 2 cm high). Note that this meant that beetles in the ‘with mites’ treatment had contact with mites only during the reproductive event (maximum of 8 days). To ensure all beetles were handled equally, we also simulated the removal of mites from beetles dispersing without mites. Adults were then fed twice a week with minced beef until they died. At this point, we recorded their life span and measured their pronotum width. This experiment was carried out in two blocks, with 45 successful replicates of beetles breeding without mites and 46 successful pairs breeding with mites. We analysed data collected only from successful breeding attempts.

### Experiment 2: Is the Change in Paternal Care Duration Adaptive?

To test whether the change in the duration of paternal care was due to adaptation or to constraint, we measured the costs associated with breeding alongside mites when males were unable to choose their time of desertion. Once again, we placed pairs of sexually mature, virgin beetles to breed on an 8–15 g dead mouse (mean carcass mass = 10.88 g, SD = 1.71) inside a plastic container (17 × 12 cm and 6 cm high) with 3 cm of soil. This time, however, parents were unable to leave the breeding box, which is standard practice when breeding burying beetles under biparental care in the laboratory (e.g. Müller, Eggert, & Elsner, 2003; Smiseth, Dawson, Varley, & Moore, 2005). We had two different treatments: beetles breeding with or without phoretic mites. As in experiment 1, for the ‘with mites’ treatment we introduced 10 deutonymphs into the breeding boxes at the same time as we introduced the mouse. We carefully placed the mites on the soil surrounding the mouse. We also weighed each carcass twice: just prior to giving it to the beetles and after it had been prepared for reproduction but before larval hatching (i.e. 56 h after pairing at the start of the experiment). In this experiment, some of our subjects were siblings from the same family. However, we controlled for this potential problem by ensuring that the genetic background of pairs was distributed evenly across treatments. For example, we paired males from family x with sisters of family y, or males from family y with females from family x, and replicated this genetic combination two to four times: at least once, although no more than twice, per mite treatment. Each genetic combination was given a unique ‘pair code’ and treated as a random effect in the analysis (see below).

As in experiment 1, 8 days after the start of the experiment we counted the larvae, weighed the brood and removed parents. We anaesthetized all parents using CO_2_, and removed and counted any mites that each of them carried before placing each adult alone in a small plastic box (12 × 8 cm and 2 cm high). Thus, as in experiment 1, this meant that beetles in the ‘with mites’ treatment had contact with mites only during the reproductive event (8 days). To ensure all beetles were handled equally, we also anaesthetized and simulated the removal of mites from beetles dispersing without mites. Adults were then fed twice a week with minced beef until they died. At this point, we recorded their life span and measured their pronotum width. We carried out this experiment in three blocks, yielding a total of 20 successful pairs breeding without mites and 23 successful pairs reproducing with mites. Only data from successful pairs were included in the analysis.

### Statistical Analysis

#### Experiment 1: success of the beetles

We performed all the analysis in the statistical program R (v. 3.0.2; R Development Core Team, 2011). We compared the departure times of males and females in each treatment using general linear mixed-effects models (lme4 package; Bates, Maechler, & Bolker, 2012). In each model we included the family of the parents as a random effect nested within the block. We included the family of the male when analysing the desertion time of the male, and the family of the female when analysing desertion time of the female. As explanatory variables we included the treatment, the mass of the carcass (either before or after preparation) and the size of the parents (or the relative size of the pair, depending on which variable explained more variance). To analyse the residual fitness of the parents we used the life span of the male (or of the female) as a response variable in general linear mixed-effects models and included the treatment, the desertion time of each sex from the nest, the size difference between the sexes (or the absolute size of males and females, depending on which variable explained more variance) and the mass of the carcass as explanatory variables. When analysing the life span of the female we included as an explanatory variable the interaction between the treatment (with or without mites) and the size difference between the parents (male size − female size). Again, we included the family of the male nested within the block when analysing male life span, and the family of the female nested within the block when analysing female life span. We analysed the success of the brood (brood size and average larval mass at dispersal) by including as fixed effects the treatment, the mass of the carcass, the size difference between the sexes (or the absolute size of males and females) and the desertion time of the parents. Because brood size and brood mass at dispersal were highly correlated in this data set (Pearson *r* = 0.91, *P* < 0.00001) we only included the size of the brood as an explanatory variable. When analysing the average larval mass we also included the total brood size as a covariate. In these models we included the experimental block as a random effect. We reduced every model using the Akaike information criterion (AIC; Akaike, 1974), and checked the distribution of the residuals from the final model. We obtained *P* values for all the models using the ‘summary’ function (Bates et al., 2012). Beetles that failed to breed were excluded from the analyses.

#### Experiment 1: success of the mites

We performed a Spearman rank correlation test to measure the association between the departure time of each parent and the number of mites dispersing with it. We analysed the success of the phoretic mites by summing the total number of mites dispersing on each parent, log transforming this value and then using this as a response variable in a general linear mixed-effects model. We included as explanatory variables the size of the male and of the female parent, the desertion time of each parent, the mass of the carcass and the size of the brood. We compared the number of mites carried by each parent and analysed whether the difference between males and females (mites on the male − mites on the female) was affected by the difference in the timing of desertion between parents (male desertion time − female desertion time). We also included as explanatory variables the size of the parents and the mass of the carcass. In every model we included the block as a random effect. We reduced every model using the AIC and checked the distribution of the residuals from the final model. We obtained *P* values for all the models using the ‘summary’ function.

#### Experiment 2

We analysed the data with general mixed-effects models (lme4 package; Bates et al., 2012), with the lmer function. In every model we included the pair code as a random effect nested within the block. To analyse offspring performance we used as response variables the size of the brood and the average larval mass. Because brood size and brood mass at dispersal were highly correlated in this data set too (Pearson *r* = 0.94, *P* < 0.00001), we included only brood size in our analysis. We included as explanatory variables the treatment, the mass of the carcass (either before or after preparation, depending on which variable explained more variance) and the size of the parents. When analysing average larval mass we also included the total brood size as a covariate. To examine the effects of our manipulations on parental life span, we analysed the data for males and females separately. When analysing parental life span we included as fixed effects the treatment, the mass of the carcass and the size of the parents (or the relative size of the parents (male size − female size), depending on which variable explained more variation). Again, when analysing the life span of the female we included as an explanatory variable the interaction between the treatment (with or without mites) and the size difference between the parents (male size − female size). We again included the pair code nested within the block as a random effect. We reduced every model using the AIC and checked the distribution of the residuals from the final model. We obtained *P* values for all the models using the ‘summary’ function.

## Results

### Experiment 1: Do Mites Change Paternal Care Duration?

#### Correlates of parental departure time

**Figure 2:**
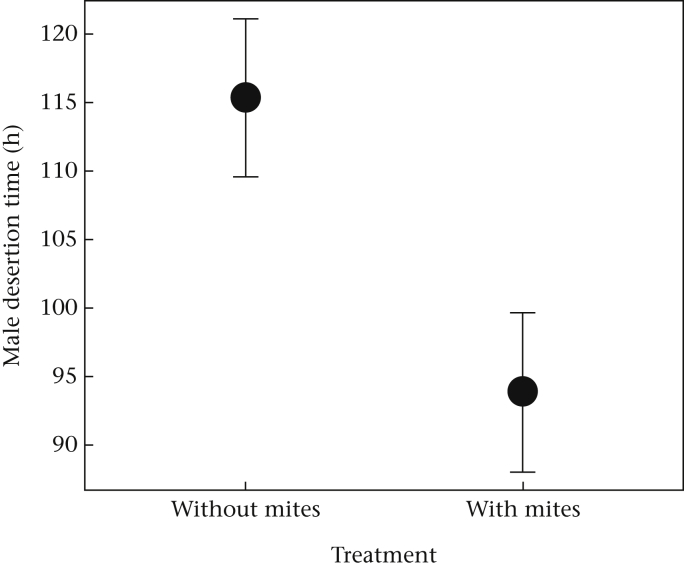
The effect of mites on the timing of male brood desertion (pairing of beetles with a mouse was at 0 h). The mean and 95% confidence intervals from the fitted values from the models are shown.

Males consistently left the brood significantly earlier than females, regardless of the presence or absence of mites (paired *t* test: *t*_151.04_ = 11.13, *P* < 0.00001). However, when mites were present, males left the brood chamber even earlier (Table 1, Fig. 2), whereas female desertion time was unchanged by the presence of the mites (Table 1). Smaller females stayed longer with their brood, and also stayed longer when the mass of the carcass was larger (Table 1).

#### Correlates of male, female and offspring fitness

**Figure 3:**
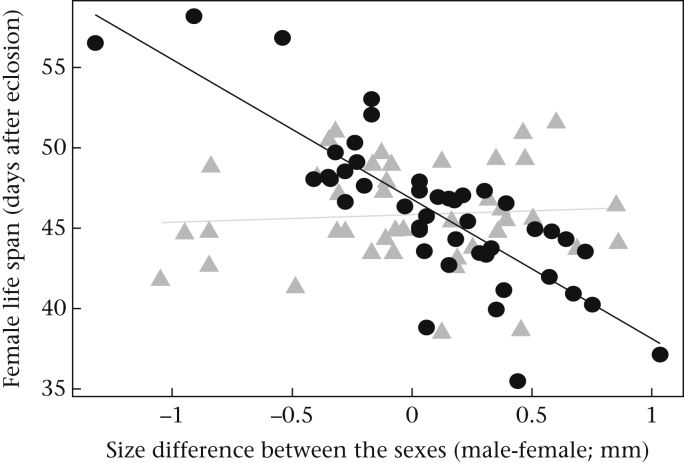
The relationship between the size difference within each pair (male − female pronotum width) and the life span of the female (measured as days after eclosion) when adult beetles were allowed to desert (experiment 1): after reproduction with mites present (black circles, black line) and in the absence of mites (grey triangles, grey line). The graph shows the linear regression between the fitted values from the model and the explanatory variable, by treatment.

Mites did not significantly change the correlates of fitness that we measured for either the offspring or the male (Table 1). We found no effect of mites on either the size of the brood, the average larval mass at dispersal or the life span of the male after reproduction (Table 1). Mites did affect the life span of the female after reproduction, but the effect was dependent on her size relative to the size of her mate (Table 1). When the mites were absent, females had a longer life span when they were smaller than their partner. When the mites were present, however, females lived for longer if they were larger than their partners (Fig. 3).

Male life span after reproduction, but not female life span, was positively correlated with the mass of the carcass given to beetles at the start of reproduction (Table 1). Brood size at dispersal and average larval mass were positively correlated with carcass mass after preparation (Table 1).

The longer the mother stayed in the nest, the heavier the offspring she produced (Table 1). Both male and female size predicted the size of the brood, with larger parents producing larger broods (Table 1).

#### Success of the mites

**Figure 4:**
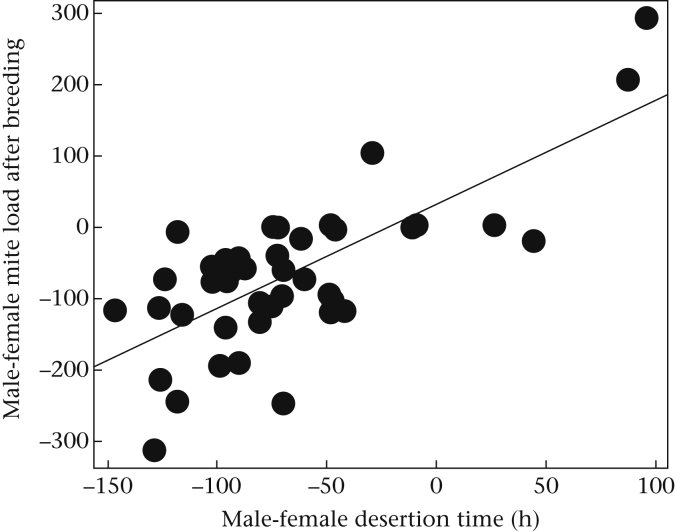
The relationship between the difference in desertion time within each pair (male − female) and the difference in mite load within each pair (male − female). The graph shows the linear regression between the raw values and the explanatory variable.

The longer beetles stayed on the carcass, the more dispersing mites they carried (males: *r*_43_ = 0.73, *P* < 0.0001; females: *r*_44_ = 0.62, *P* < 0.0001). Therefore, when the male left earlier, the female dispersed with relatively more mites than her partner (Table 2, Fig. 4). The longer the female stayed in the nest, the more mites dispersed in total from the nest. Carcass mass (*P* = 0.70), male size (*P* = 0.39) and female size (*P* = 0.68) were not significantly related to the number of mites dispersing on the parents.

### Experiment 2: Is the Change in Paternal Care Duration Adaptive?

**Figure 5:**
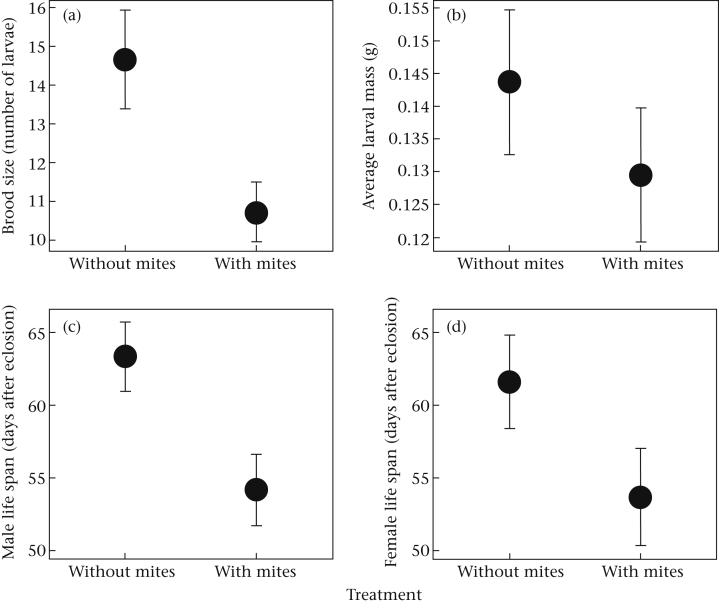
The effect of mites on (a) brood size at dispersal, (b) the average larval mass, (c) male life span after reproduction and (d) female life span after reproduction. The mean and 95% confidence intervals from the fitted values from the models are shown.

**Figure 6:**
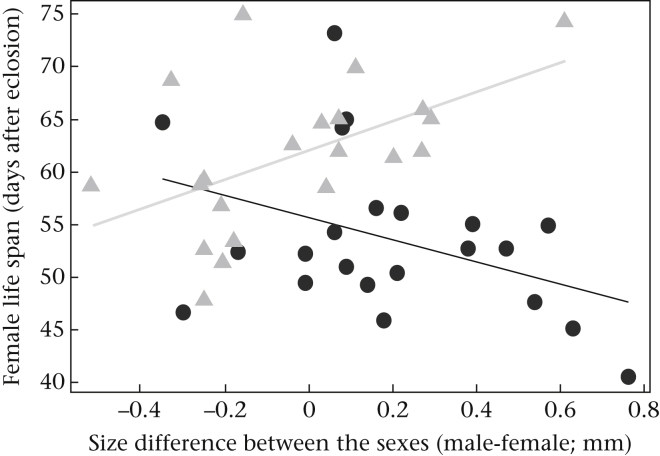
The relationship between the size difference within each pair (male − female pronotum width) and the life span of the female (measured as days after eclosion) when adult beetles were prevented from deserting (experiment 2): after reproduction with mites present (black circles, black line) and in the absence of mites (grey triangles, grey line). The graph shows the linear regression between the fitted values from the model and the explanatory variable by treatment.

When males were prevented from leaving the brood, the presence of mites caused every member of the family to suffer a loss in fitness. Broods were smaller when mites were present (Table 3, Fig. 5a) and larvae were on average lighter (Table 3, Fig. 5b). In the presence of mites, males had a shorter life span (Table 3, Fig. 5c), as did females (Table 3, Fig. 5d). Once again, mites influenced female fitness, but the effect was dependent on the female's size relative to her partner's (Table 3). As in experiment 1, when mites were absent, females had a longer life span when they were smaller than their partner. When the mites were present, however, females lived for longer if they were larger than their partners (Fig. 6).

The mass of the carcass at the start of reproduction was positively related to male life span (Table 3), whereas the mass of the prepared carcass was positively related to female life span (Table 3). Larger females produced smaller broods (Table 3).

## Discussion

Our experiments on burying beetles show that interactions with a second species can change the extent of paternal care, and in a way that is adaptive for the male. We showed that phoretic mites of the *P. carabi* species complex increased the costs associated with prolonged paternal care in the burying beetle. When breeding in the presence of phoretic mites, male beetles spent less time with their larvae after hatching, and in so doing enhanced their offspring's fitness (i.e. offspring number and body mass) and their own residual fitness (i.e. life span). By flexibly adjusting their desertion time in response to mite infestation, males ensured that their fitness was not compromised by the mites. We suggest that variation in male desertion time could be regarded as an adaptive reaction norm that might vary flexibly in response to the extent of mite infestation.

In burying beetles, biparental care is thought to have evolved to promote reproductive success in the face of intense interspecific competition for a rare, but valuable breeding resource (Robertson, 1993, Scott, 1989, Scott, 1998, Trumbo, 1991, Trumbo, 1994). Yet, males rarely stay until their young disperse from the nest (Scott, 1998). Our experiments suggest that there are costs, as well as the previously identified benefits, associated with prolonged paternal care, and that variation in these costs might account for variation in the duration of male care. Previous studies have shown that if males abandon the nest too soon, then they risk losing their breeding resource, or their brood, to a *Nicrophorus* rival (Scott, 1989, Scott, 1998, Trumbo, 1991, Trumbo, 1994). This study shows that by staying too long when there are phoretic mites breeding alongside them on the carcass, males risk reducing their fitness, and also have to carry a larger number of mites as they fly off. Thus, the duration of paternal care in burying beetles is apparently influenced by multiple interspecific interactions, some of which promote the duration of paternal care, while others act to curtail it. Under more natural conditions than we created in our experiments, where a greater number of species interact with the burying beetle family, the magnitude of the costs imposed by mites could be rather different. For example, perhaps *P. carabi* pose less of a threat to burying beetle fitness when there are other heterospecific rivals for the carcass, such as blowflies, nematodes or fungi, because mites feed upon these species instead of the carrion (e.g. Springett, 1968, Wilson, 1983, Wilson and Knollenberg, 1987), so incidentally benefiting the burying beetle through a by-product mutualism.

Why exactly did the mites pose such a threat to fitness for the different members of the family in our second experiment? The most likely explanation is that mites and burying beetles are in competition to exploit the resources on the carcass. Each member of the burying beetle family consumes the carrion, and the parents are able to recoup some of their costs of reproduction in this way (Boncoraglio and Kilner, 2012, Creighton et al., 2009, Scott, 1998). This is probably why the size of the carcass is positively correlated with residual adult reproductive success, as well as with the number of offspring produced. When we experimentally forced males to remain on the carcass alongside mites, we intensified this competition and so reduced the fitness of male and female burying beetles (because the size of the brood, average larval mass and the life span of the male was reduced when the mites were present). Whether mites increase the costs of prolonged paternal care to males directly or whether costs are increased through intensified conflict with the female over resources on the carcass (Boncoraglio & Kilner, 2012) is not possible to disentangle from these experiments. Nevertheless, it is likely that mites influence competitive interactions between males and females on a carcass. We know that females recoup the costs of reproduction by consuming the carrion, and that when the male is there he apparently competes with her over this carrion (see Boncoraglio & Kilner, 2012). We found that when females were larger than their partners, and therefore competitively dominant (e.g. Otronen, 1988), they had greater residual fitness than females that were smaller than their partner, but only when competition for resources on the carcass was intensified by the presence of mites (Figure 3, Figure 6). Perhaps mites could have influenced how males and females divide resources on the carcass to recoup their costs of reproduction, somehow preventing relatively small, competitively inferior males from taking as much as they do when mites are absent.

Offspring fitness was also reduced in the presence of mites because broods were smaller at dispersal. Smaller broods might have arisen because mites consumed some of the beetle's eggs, as has been reported in previous work (Beninger, 1993, Blackman and Evans, 1994). Furthermore, partial filial cannibalism may have yielded fewer young (Bartlett, 1988), either because competition with mites forced parents to consume their own larvae or because mites reduced the value of the current breeding attempt, and consequently induced partial filial cannibalism (Bartlett, 1988). Although these two alternatives are distinct, they are not mutually exclusive. In the former, parents invest in future reproduction by consuming some of their current brood to increase their residual fitness whereas in the latter, investment in future reproduction is achieved by reducing the costs of current brood care. However, whether or not mites really do induce filial cannibalism remains to be determined. Furthermore, larval mass was also smaller when the mites were present, even though the broods were smaller. What accounts for this result remains to be addressed, but one possibility is that parents reduce the extent of care when there are mites present, thus consuming more of the carrion themselves rather than providing food for their offspring.

An interesting by-product of the males' earlier desertion time is that it potentially creates a new form of sexual conflict, centred on the transport of phoretic mites away from the breeding event. Just as honeybees, *Apis mellifera*, sustain greater energetic costs when they fly loaded with pollen compared with when they fly unburdened (Wolf, Schmid-Hempel, Ellington, & Stevenson, 1989), so it is likely that the transport of mites is not entirely cost-free. Indeed, beetles have been observed to arrive at a carcass with 100 mites on them (Scott, 1998), which is equivalent to approximately half the burying beetle's mass. By leaving the current breeding attempt earlier, the male not only spares his offspring's fitness, and defends his own residual fitness, he also departs before most mites have completed development, and so takes fewer mites with him. This leaves the female carrying many more mites with her when she eventually departs, so enabling males to win any sexual conflict over mite transport.

To sum up, our experiments show that interactions with other species change the costs associated with paternal care in burying beetles, probably by increasing competition for resources, and this may either directly or indirectly affect the sexual conflict between parents. Males respond adaptively by changing the length of time they spend with their brood. Our experiments thus show how interspecific interactions can account for some of the intraspecific variation seen in nature in the duration of male care. Whether similar patterns can be found in other species remains an exciting challenge for future work.

## Figures and Tables

**Table 1:** Results from the final models for each variable analysed in experiment 1

Factor	Estimate	SE	*df*	*t*	*P*
**Male desertion time**					
Carcass mass after preparation	1.83	2.76	84.74	0.66	0.50
Mites present	−18.65	8.79	84.10	−2.12	0.036*
**Female desertion time**					
Carcass mass after preparation	6.48	1.8	84.70	3.6	0.0005*
Female size	−23.56	10.54	72.93	−2.23	0.02*
**Brood size**					
Carcass mass after preparation	1.75	0.49	82.34	3.57	0.0005*
Size of the male	9.13	2.42	82.47	3.76	0.0003*
Size of the female	6.46	2.70	82.88	2.38	0.01*
**Average larval mass**					
Carcass mass after preparation	0.006	0.001	85	4.85	<0.0001*
Brood size	−0.002	0.0002	85	−9.00	<0.0001*
Female desertion time	0.0002	0.00007	85	3.57	0.0005*
**Male life span**					
Carcass mass before preparation	1.52	0.66	85.99	2.28	0.02*
Size of the male	−7.07	3.83	81.10	−1.84	0.06
**Female life span**					
Treatment	0.90	1.93	64.97	0.47	0.64
Size difference between parents (male−female)	0.60	3.17	83.35	0.19	0.84
Size difference between parents (male−female)*Treatment	−9.36	4.38	74.39	−2.13	0.03*

*N* = 45 for the ‘without mites’ and *N* = 46 for the ‘with mites’ treatment for the success of the brood and for the desertion time of the female, *N* = 45 for each treatment for the life span of the female, *N* = 43 for the ‘without mites’ and *N* = 46 for the ‘with mites’ treatment for the life span of the male and *N* = 45 for each treatment for the desertion time of the male. The initial models included the mass of the carcass (either before or after preparation) and the size of the parents (or the relative size of the parents (male size − female size)) as explanatory variables. We reduced models using the AIC (Akaike, 1974).

*P < 0.05.

**Table 2:** Results from the final models for the difference in mites dispersing on each parent and the total number of mites dispersing on both parents in experiment 1

Factor	Estimate	SE	*df*	*t*	*P*
**Final number of mites dispersing on both parents (male+female, log transformed)**					
Female desertion time from the nest	0.01	0.002	42	4.70	<0.0001*
Male desertion time from the nest	0.0003	0.001	42	2.18	0.03*
**Difference in mites dispersing on each parent (male−female)**					
Difference in desertion time (male−female)	1.61	0.20	40.70	7.8	<0.0001*
Size difference between parents (male−female)	36.97	24.69	40.66	1.49	0.14
Carcass mass before preparation	−9.4	5.86	40.34	−1.62	0.11

*N* = 45. The initial models included as explanatory variables the size of the male and of the female parent, the mass of the carcass and the size of the brood. We reduced models using the AIC (Akaike, 1974).

*P < 0.05.

**Table 3:** Results from the final models for each variable analysed in experiment 2

Dependent variable	Factor	Estimate	SE	*df*	*t*	*P*
Brood size	Treatment	−4.30	2.06	35.5	−2.08	0.04*
Size of the female	−10.05	4.62	33.61	−2.17	0.03*
Average larval mass	Treatment	−0.02	0.008	37.2	−2.8	0.007*
Brood size	−0.002	0.0006	38.55	−4.69	<0.0001*
Carcass mass after preparation	0.007	0.003	25.26	2.45	0.02*
Male life span	Treatment	−9.08	2.68	32.61	−3.3	0.001*
Carcass mass before preparation	2.06	0.88	39.72	2.35	0.02*
Female life span	Treatment	−7.33	2.21	28.35	−3.3	0.002*
Size difference between parents (male−female)	21.56	6.19	32.9	3.4	0.001*
Carcass mass after preparation	2.72	0.79	18.09	3.4	0.003*
Size difference between parents (male−female)*Treatment	−23.48	7.64	29.62	−3.07	0.004*

*N* = 21 for the ‘with mites’ and *N* = 20 for the ‘without mites’ treatment for all the models. The initial models included the mass of the carcass (either before or after preparation) and the size of the parents (or the relative size of the parents (male size − female size)). We reduced models using the AIC (Akaike, 1974).

*P < 0.05.
